# Extracranial Nasosinusal Meningioma: A Case Report of a Rare Entity

**DOI:** 10.1007/s12070-022-03462-x

**Published:** 2023-01-06

**Authors:** Antonio Romano, Francesco Maffia, Vincenzo Iaquino, Giuseppe Tarallo, Vincenzo Abbate, Gianluca Renato De Fazio, Umberto Committeri, Paola Bonavolontà, Luigi Califano, Giovanni Dell’Aversana Orabona

**Affiliations:** Maxillofacial Surgery Unit, Department of Neurosciences, Reproductive and Odontostomatological Sciences, University of Naples “Federico II”, Via Sergio Pansini 5, 80131 Naples, Italy

**Keywords:** Extracranial meningioma, Nasosinusal meningioma, Endoscopis sinus surgery, Neurosurgery, Endoscopic approach

## Abstract

Meningiomas are the most common neoformations of the central nervous system, and represent the 33% of all intracranial neoplasms. The nasosinusal tract is involved in 24% of cases of extracranial localization. The aim of our paper is to present the case of a patient with an ethmoidal sinus meningioma.

## Introduction

Meningiomas are the most common neoplasms of the central nervous system [1]. These tumors represent approximately the 33% of all intracranial neoplasms [2]. They can originate from meningeal cells, subarachnoid blood vessels, fibroblasts and pia mater [1]. Extracranial Meningiomas (EMs) are very rare, with a incidence of less than 2% [3]. In particular, among the extracranial localizations, the nasosinusal tract is involved in only 24% of cases [4]. Commonly, meningiomas do not have a tendency to infiltrate the surrounding structures but have a slow expansive evolution [5]. The symptoms are very variable as it depends on the intracranial structures that are compressed or occupied by the mass [2]. In the nasal tract most referred symptoms can be described as: perception of nasal obstruction, epistaxis, sinusitis, anosmia, intermittent headaches, and facial pain [3]. The aim of our paper is to present the case of a patient with a past medical history positive for recurrent transitional meningiomas, with recent evidence on MRI of a neoformation in the ethmoidal sinus, and treated with endoscopic endonasal approach.

## Case Report

### Preoperative Findings

The study was conducted in accordance with the Declaration of Helsinki. In December 2021, a 29-year-old female patient came to the Authors’ observation with current evidence on MRI of a neoformation in the ethmoidal sinus. The mass maximum diameter was 2.8 cm. The patients referred a remote medical history of Acute Lymphoid Leukemia (ALL) and two episodes of transitional meningiomas in the posterior fossa. ALL was first of diagnosed in February 1996, and was initially treated with intensive chemotherapy according to the AIEOP LAL 9502 protocol. In June 1997 the disease relapsed, and the patients was treated with total body irradiation + chemotherapy (Cyclophosphamide and Etoposide) with subsequent umbilical cord hematopoietic stem cell (HSC) transplantation. In April 2016 she complained of reduced sensitivity in the left cheek and left lip, and a brain CT showed voluminous expansive formation in the cistern of the left pontocerebellar corner. For this reason, she underwent right frontal ventriculostomy, left suboccipital craniotomy and removal of the neoplasm which was reported as a “transitional meningioma” (WHO Grade I). In March 2018 an expansive extra-axial mass with lateral subtentorial site attached to the tentorium and the cerebellar dura was found during a follow-up brain MRI. Few weeks later, the patient underwent left retromastoid craniotomy surgery for microsurgical removal a tentorial mass. The histopathological examination report diagnosed transitional meningioma (WHO Grade I). The patient, during regular postoperative follow-ups, continued to execute MRI and CT of the brain.

### Surgical Treatment

**Fig. 1:**
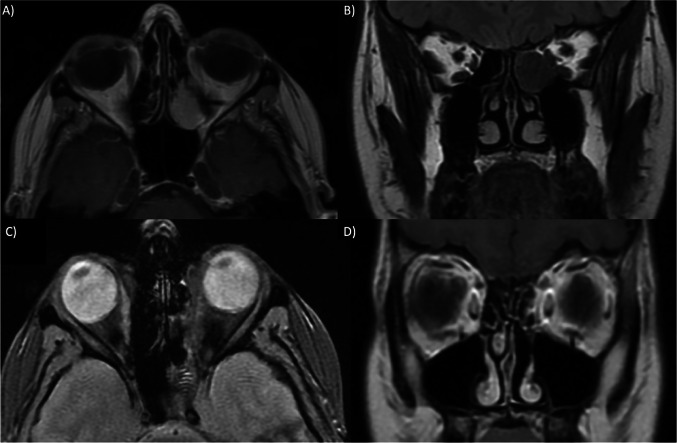
Preoperative imaging A, B: **A** MRI in T1 showing an ethmoidal mass in axial view; **B** Same MRI but in coronal view showing a mass attached to the lamina papyracea. Postoperative 6 months follow-up imaging C, D: **C** MRI in T2 showing surgical results in axial view; **D** Same MRI but in coronal view showing the absence of ethmoidal mass

Considering the mass localization and dimension, the patient was planned for endoscopic endonasal surgery for tumor removal (Fig. 1A, B).

The surgical treatment consisted of a combination between Endoscopic Sinus Surgery (ESS) + tumor removal by debulking technique, aiming the macroscopic radicality. The surgical procedure was performed under general anesthesia using a 4-mm, 0°–30° endoscope. Cottonoids soaked with diluted.

**Fig. 2:**
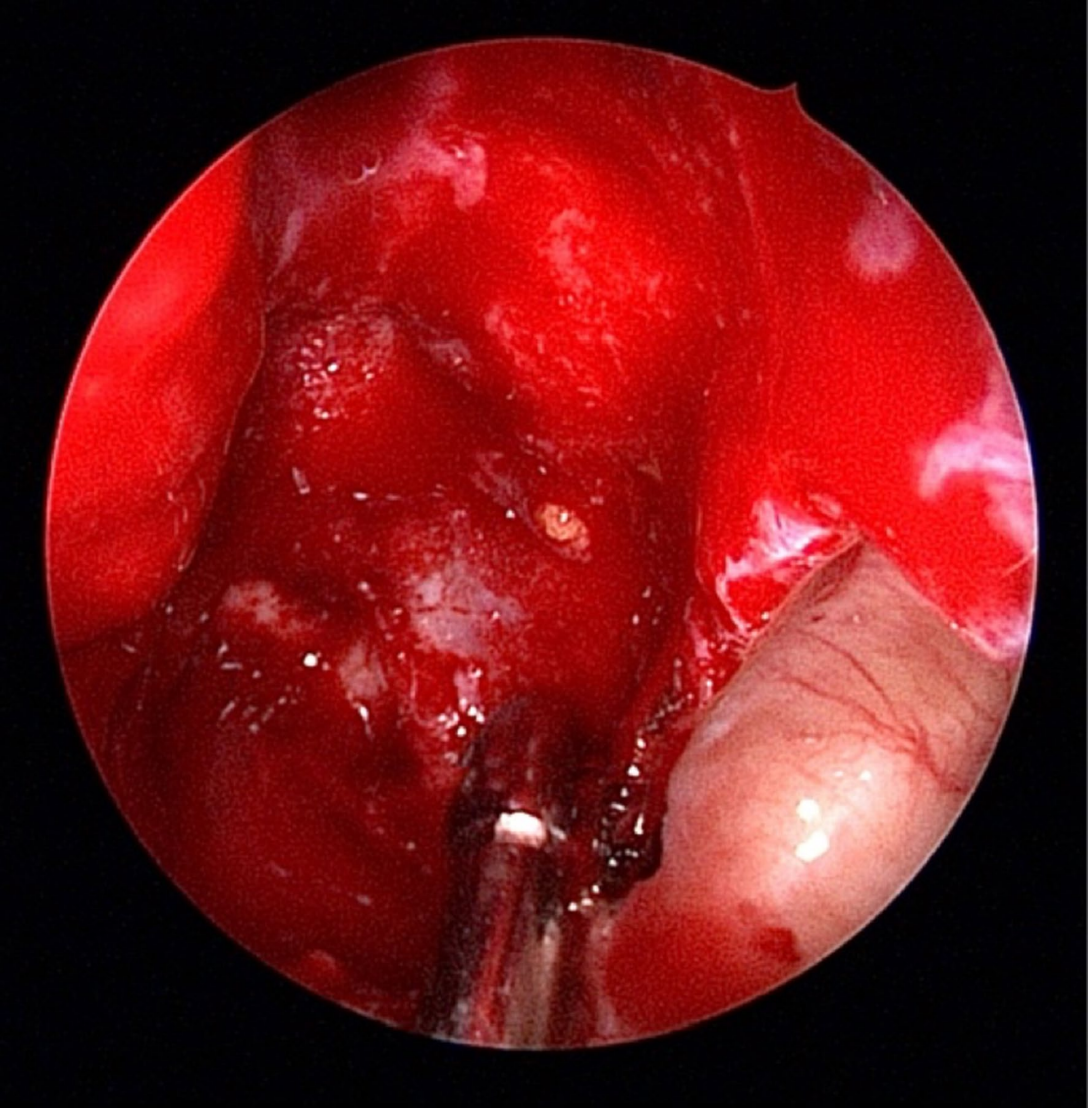
Intraoperative endoscopic visualization of the ethmoidal mass and its relationship with surrounding structures

Epinephrine (1:100,000) were filled in the middle meatus. The middle turbinate is gently medialized to improve the operating field. A horizontal incision was performed over the uncinate process, allowing a better exposition. An anterior ethmoidectomy was performed by removing the ethmoidal bulla to individuate the mass, that appeared attached to the lamina papyracea. The middle turbinate was crossed in its second portion, the lamina basalis, finally showing the mass. The neoplasm appeared pearly, with a hard elastic consistency but easily cleaved (Fig. 2).

The debulking was performed removing the mass by dividing it in pieces. The lamina papyracea was dissected from the mass with great attention to its integrity. At the end of the surgery, the sphenoid sinus ostium and the lamina cribrosa roof were checked for possible dissemination and the middle turbinate tail was gently removed. A careful debridement of the surrounding bone was performed to increase the mascroscopic radicality. One merocel was positioned in the nasal fossa. The postoperative phase was free from complications. After 3 days the merocel was removed and the patient was discharged from hospital.

### Postoperative Findings

The histopathological examination described the mass as a meningioma psammomatous (WHO Grade I). The patient underwent regular follow-ups by endoscopic endonasal examination, without signs and symptoms of a relapse. A MRI was requested at 6 months from the surgical treatment showing no signs of relapse or presence of new masses (Fig. 1C, D).

## Discussion

Meningiomas are mostly benign tumors originating from non-neuroepithelial progenitor cells, the cells of the arachnoid cap [6]. The possible risk factors contributing to the onset of these tumors are various and not always ascertained, including: the deletion in the NF2 gene, cranial ionizing radiation, and progesterone exposition [7]. In Literature, the association between radiation-induced meningiomas in patients with acute lymphoid leukemia is already described: the whole-brain irradiation used for the hematological disease seems to be the stimulus to develop multiple and asynchronous meningeal lesions [8].

WHO described three grades for Meningiomas classification: Grade 1, benign meningiomas; Grade 2, atypical meningiomas; Grade 3, malignant meningiomas with anaplastic and invasive pattern [9]. To be defined as atypical, a meningioma must be characterized by intense mitotic activity (Ki-67 > 5%). In our case, the histopathological examination determined Grade I, with (Ki-67 < 5%). Meningiomas are also classified in different histopathological subtypes: transitional, psammomatous, meningothelial, fibroblastic, syncytial, and mixed forms [9].

Among all cases of meningiomas with extracranial localization involving the nasosinusal tract, the paranasal region has the tendency to be affected in more than one sinus [4]. The endoscopic endonasal approach represents the gold standard in the treatment of extracranial nasosinusal meningioma for its many benefits: direct and wide vision of the surgical field, multi-angled visualization, and scar-less surgery with minimal invasiveness. In particular, considering the necessity to remove the attachments, a close-up visualization improves the macroscopic radicality [4].

The long-term prognosis of meningiomas is based on two factors: the histological grading of the tumor and the extent of the resection [9]. In 1957, Simpson developed a scale to classify the degree of surgical resection: Grade 1 excision is characterized by the complete removal of the tumor, its dural attachment and any invaded bone; Grade 2 procedure includes tumor removal with any visible extensions and coagulation of the dura mater; Grade 3 excision involves macroscopic removal of the tumor without resection of the dural attacks and extradural extensions, such as invasion of the surrounding bone [10]. Grades 1 to 3 resection describe a gross total resection (GTR); Subtotal resection (STR) would represent Grade 4 with partial excision, while Grade 5 consists of simple decompression without removal of the mass [10]. Our patient underwent a GTR with endonasal endoscopic approach for the removal of the neoformation in the ethmoid region. In our case no recurrence was observed and MRI showed normal pneumatization of the previously affected sinus.

The therapeutic target of meningiomas is the total removal of the neoformation and a careful, close and long follow-up to monitor the appearance of relapses or relics. Histopathological examination with particular attention to Ki-67% is the key to choose the most valuable treatment depending on the risk of malignancy [9]. Our study showed how, according to the Literature, the endoscopic endonasal approach represents a valid option in the treatment of nasosinusal meningiomas.
